# MiR-182-5p Modulates Prostate Cancer Aggressive Phenotypes by Targeting EMT Associated Pathways

**DOI:** 10.3390/biom12020187

**Published:** 2022-01-22

**Authors:** Marilesia Ferreira Souza, Ilce Mara Syllos Cólus, Aline Simoneti Fonseca, Valquíria Casanova Antunes, Deepak Kumar, Luciane Regina Cavalli

**Affiliations:** 1 General Biology Department, State University of Londrina, Londrina, PR 86057-970, Brazil; marilesiah@gmail.com (M.F.S.); ilcecolus@gmail.com (I.M.S.C.); 2 Department of Oncology, Lombardi Comprehensive Cancer Center, Georgetown University, Washington, DC 20007, USA; 3 Research Institute Pelé Pequeno Príncipe, Faculdades Pequeno Príncipe, Curitiba, PR 80240-020, Brazil; aline.fonseca@pelepequenoprincipe.org.br (A.S.F.); valquiria.antunes@aluno.fpp.edu.br (V.C.A.); 4 Department of Pharmaceutical Sciences, North Carolina Central University, Durham, NC 27707, USA; dkumar@nccu.edu

**Keywords:** prostate cancer, miRNA, miR-182-5p, tumor phenotypes, EMT

## Abstract

Prostate cancer (PCa) is a clinically heterogeneous disease, where deregulation of epigenetic events, such as miRNA expression alterations, are determinants for its development and progression. MiR-182-5p, a member of the miR-183 family, when overexpressed has been associated with PCa tumor progression and decreased patients’ survival rates. In this study, we determined the regulatory role of miR-182-5p in modulating aggressive tumor phenotypes in androgen-refractory PCa cell lines (PC3 and DU-145). The transient transfection of the cell lines with miR-182-5p inhibitor and mimic systems, significantly affected cell proliferation, adhesion, migration, and the viability of the cells to the chemotherapeutic agents, docetaxel, and abiraterone. It also affected the protein expression levels of the tumor progression marker pAKT. These changes, however, were differentially observed in the cell lines studied. A comprehensive biological and functional enrichment analysis and miRNA/mRNA interaction revealed its strong involvement in the epithelial-mesenchymal transition (EMT) process; expression analysis of EMT markers in the PCa transfected cells directly or indirectly modulated the analyzed tumor phenotypes. In conclusion, miR-182-5p differentially impacts tumorigenesis in androgen-refractory PCa cells, in a compatible oncomiR mode of action by targeting EMT-associated pathways.

## 1. Introduction

Prostate cancer (PCa) is the most incident tumor and the fifth cancer-related death in men worldwide, with approximately 1.4 million cases diagnosed and 375,000 deaths per year [1]. PCa can be diagnosed as local or advanced, with metastasis development into other organs and bones [2,3]. The emergence of more sensitive imaging methods and diverse treatment options, including radiotherapy, radical prostatectomy, and androgen deprivation therapy, have increased the survival rates, particularly in primary PCa cases. However, the molecular complexity and heterogeneity of this tumor is a challenge that continues to impair early diagnosis, treatment selection, and prediction of treatment resistance, and clinical outcome [2]. Significant advances have been achieved in the analysis of molecular markers that underlie the diverse stages of PCa tumorigenesis, contributing to the characterization of the genomic landscape of these tumors that can be translated to clinical practice [4].

Epigenetic markers, such as miRNAs, have emerged as powerful molecular markers that play a pivotal role in regulating mRNA targets involved in biological pathways commonly affected in cancer cells, such as the ones that control cell proliferation and differentiation, cell cycle, apoptosis, migration, and invasion [5,6]. MiRNAs were shown to be dysregulated in PCa, among them the highly conserved miR-183 family. This family is composed of three members, miR-96, miR-182, and miR-183, mapped at 7q32.2, which up-regulation has been reported in PCa clinical cases [7,8]. MiR-182-5p specifically, has been shown to be overexpressed in PCa tissues [8,9,10,11,12] in association with tumor progression and decrease in survival rates [10,13,14,15]. In PCa cell models, deregulation of miR-182-5p has been shown to significantly impact cell proliferation, colony formation, migration, and invasion [12,14,16,17,18].

Although these functional in vitro studies provided compelling evidence of the involvement of miR-182-5p in PCa tumorigenesis, they yielded contradictory results in different tumors’ cell models, and in some cases, even within the same tumor type. Therefore, in this study, our main goal was to determine the role of miR-182-5p in modulating cancer aggressive phenotypes, such as increased cell proliferation, adhesion and migration capabilities, and treatment resistance in the androgen-refractory PCa cell lines PC-3 and DU145. Changes in these phenotypes were observed in the cell lines studied upon miR-182-5p inhibition and/or ectopic expression, mostly compatible with an oncomiR mode of action. However, manipulation of the endogenous miR-182-5p expression levels differentially affected the analyzed phenotypes in the PCa cells: the ectopic expression of miR-182-5p significantly increased cell proliferation, and cell migration in the PC-3 cells; inhibition of its levels, changed the adhesion time of the cells to the plates’ substrate and the down-regulation of the tumor progression marker pAKT. In the DU145 cells, the ectopic expression of miR-182-5p increased resistance to docetaxel, and the inhibition led to increased resistance to abiraterone and the decrease of the cells proliferation rate. A comprehensive biological and functional enrichment analysis and miRNA/mRNA target interaction revealed the strong involvement of miR-182-5p in regulating mRNA targets of the epithelial-mesenchymal transition (EMT) associated pathways. Protein and mRNA expression analysis of EMT markers in the miR-182-5p transfected PC-3 cells were identified: up-regulated expression levels of ZEB1, N-cadherin, Vimentin, and Claudin 1 and down-regulated expression of the EMT repressors, SNAIL1 and SNAIL2 were observed with the ectopic expression of miR-182-5p; in the transfected cells with the miR-182-5p inhibitor, down-regulated expression levels of E-cadherin and β-catenin were observed.

## 2. Materials and Methods

### 2.1. Prostate Cancer Cell Lines

The androgen-refractory PCa cell lines (PC-3 and DU145) were obtained from the Tissue Culture Shared Resource (TCSR), Lombardi Comprehensive Cancer Center, Georgetown University (Washington, DC, USA). Prior to miRNA analysis, all the lines were authenticated by short tandem repeat (STR) profiling assays, following the International Cell Line Authentication Committee (ICLAC) guidelines [19]. Cells were cultured in a 5% CO_2_ humidified incubator at 37 °C, in RPMI 1640 supplemented with antibiotics (Invitrogen, Carlsbad, CA, USA) and 10% of fetal bovine serum (FBS).

### 2.2. RNA Isolation and MiR-182a-5p Expression Analysis

RNA was isolated from PC-3 and DU145 cell lines using mirVana miRNA isolation kits (Ambion, Austin, TX, USA), following the manufacturer’s instructions. RNA quantification was performed in the spectrophotometer nanodrop 2000 (Thermo Fisher Scientific, Wilmington, DE, USA). Mature miR-182-5p expression was analyzed by Taqman miRNA assay (ID: 002334) by RT-qPCR using miRNA RNU48 (ID: 001006) as a reference in the mean value of the three-cycle thresholds.

### 2.3. MiR-182-5p Transfection Assays

The PC-3 and DU145 cell lines were reverse transfected by Lipofectamine^®^ RNAiMAX (Invitrogen, Carlsbad, CA, USA) following the manufacturer’s instructions, using has-miR-182-5p mirVanaTM miRNA inhibitor (ID:MH12369) and mimic (ID:MC12369) assays (AMBION, Austin, TX, USA). MirVanaTM miRNA inhibitor let-7c was used as a positive control (PC) and mirVanaTM miRNA inhibitor as negative control (NC). Inhibition and overexpression of miR-182-5p were achieved using a final concentration of 50 nM and 10 nM, after 48 and 24 h of transfection, respectively. The expression of the HMGA2 protein was evaluated by Western Blot to check transfection effectiveness. All transfections were performed in triplicate independent experiments and verified for efficiency prior to each functional assay.

### 2.4. Cell Proliferation Assays

Approximately 3 × 10^3^ PC-3 and DU145 cells were reversed transfected with miR-182-5p inhibitor and mimic assays, in 96-well plates and exposed to Cell Titer 96^®^AQueous One Solution (Promega, Madison, WI, USA). The proliferation curves were determined at 24, 48, and 72 h after transfection, in relation to the negative control (NC), by measuring the 490 nm absorbance at an ELISA reader (Bioteck, Winooski, VT, USA). Independent triplicate experiments were performed, each in technical triplicate.

### 2.5. Cell Cycle Assays

PC-3 cells and DU145 cells were transfected with both inhibitor and mimic miR-182-5p assays, in six-well plates at 2 × 10^5^ cells/well and fixed in absolute alcohol for cell cycle analysis. The cells were stained with propidium iodide and analyzed on FACSAria system utilizing FACSDiva and FCS Express 4 software (DeNovo Software, Los Angeles, CA, USA) with peripheral blood lymphocyte as an internal control.

### 2.6. Cell Adhesion Assays

The PC-3 and DU145 transfected cells were analyzed for cell adhesion using the xCELLigence real-time cell analyzer system (ACEA Biosciences Inc., San Diego, CA, USA). Approximately 3 × 10^3^ of the cells were placed in E-plates, previously equilibrated with 50µL of RPMI at room temperature for 30 min, according to the manufacturer’s instructions. The analysis was performed from 0 to 3 h of experiment, according to Kho et al. [20]. The Cell Index units were obtained from xCELLigence Software at each 15 min and represented as one point in the curve. The adhesion index was determined in relation to the negative control (NC).

### 2.7. Cell Migration Assays

Cellular migration activity was evaluated by standard wound-healing assays, using Culture-Insert (Ibidi GmbH, Gräfelfing, Germany). Approximately 1.2 × 10^4^ of PC-3 and DU145 cells were transfected into each side of the inserts. After transfection time, the inserts were removed, and the cells were washed with PBS to allow cell migration. A low concentration (2%) of FBS was used, as recommended to suppress cell proliferation in wound healing assays [21,22]. Photomicrographs were taken in 0 h and after 6, 24, and 48 h and the gap distance between the inserted sides was measured in pixels by ImageJ [23].

### 2.8. Cell Viability Assays

Cell viability assays were performed using MTT assay (3-(4,5-Dimethylthiazol-2-yl)-2,5-Diphenyltetrazolium Bromide) (Thermo Fisher Scientific, Eugene, OR, USA) following the manufacturer’s instructions. Approximately 3 × 10^3^ PC-3 and DU145 cells were transfected with miR-182-5p inhibitor and mimic assays in 96-well plates and treated with Docetaxel (Sigma-Aldrich, Thermo Fisher Scientific, Eugene, OR, USA) and/or Abiraterone (Sigma-Aldrich, Thermo Fisher Scientific, Eugene, OR, USA) in the concentrations of 1 nM, 100 nM, and 1000 nM. The most optimal concentrations for each cell line were previously tested in parental cells (not transfected). The absorbance was read at 540 nm in an ELISA reader (Bioteck, Winooski, VT, USA), after 72 h.

### 2.9. Computation Analysis of MiR-182-5p Biological Function and Pathway Analyses, and Interaction with mRNA Targets of the EMT Process

Diana miRPath v.3.0 [24], was used to assess the biological pathways regulated by miR-182-5p. Enrichment of Kyoto Encyclopedia of Genes and Genomes (KEGG) pathways among miR-182-5p target genes were analyzed, and pathways with *p*-value < 0.05 (FDR corrected) were considered significant. miRTarBase [25] and TarBase v. 8 [26] were used to identify interactions between the miR-182 family (miR-182-5p, miR-182-3p, miR-183-5p, miR-183-3p, miR-96-5p, miR-96-3p) and their experimentally validated target genes. Only interactions validated based on strong assays (Reporter assay, Western blot, and RT-qPCR) were considered. The target genes for miR-182-5p observed simultaneously in the two databases were “cross-checked” with genes related to EMT function from the dbEMT2 [27], which collect experimentally verified EMT-related genes from literature, and the EMTRegulome databases [28]. The EMTRegulome program, which provides regulatory relationships of several types of regulators and targets based on co-regulation motifs for the EMT process, was also used to identify the regulatory relationships of miR-182-5p with factors of transcription (TF) and other mRNA targets. The motif type 1, and a *p* value < 0.05 were the criteria established for this analysis. The STRING v.11 program [29], was used to verify the protein-protein interactions (PPI) of the identified EMT-associated mRNA targets. Finally, Cytoscape v.3.8.0 [30], was used to construct molecular interaction networks of miR-182-5p selected EMT mRNA targets and the EMT markers evaluated in this study (*CDH1, CDH2, CLDN1, CTNNB1, SNAI1, VIM,* and *ZEB1*).

### 2.10. EMT Markers Expression Analysis

The expression of EMT markers was assessed by both protein and mRNA levels, by Western Blot and RT-qPCR, respectively. For protein expression, PC-3 and DU145 transfected cells were lysed using RIPA buffer with Protease and Phosphatase Inhibitor Mini Tablets (Pierce Biotech., Rockford, IL, USA). The protein concentrations were measured using Pierce™ BCA Protein Assay Kit (Pierce Biotech., Rockford, IL, USA) following manufactures’ recommendations. Total protein (40 μg) (and the reference protein) was subjected to Bolt Mini GelsTM (Thermo Fisher Scientific, Eugene, OR, USA), using the iBlot 2 Gel Transfer Device (Thermo Fisher Scientific) followed by immunoblotting. The proteins were visualized using SuperSignal ECL (Thermo Fisher Scientific). The primary antibodies used were: Epithelial-Mesenchymal Transition (EMT) (Antibody Sampler Kit, Cell Signaling Technology, Inc., Danvers, MA, USA; dilution 1:1000, except ZEB1: dilution 1:200), HMGA2 (Cell Signaling Technology, Inc.; dilution 1:750), p-AKT (Cell Signaling Technology, Inc.; dilution 1:1000), and GAPDH (Cell Signaling Technology, Inc.; dilution 1:2000). The gel bands were quantified using ImageJ software [23].

Gene expression of the EMT markers were performed for the *CDH1* (Hs01023894_m1), *CDH2* (Hs00983056_m1), *CLDN1* (Hs00221623_m1), *CTNNB1* (Hs00355049_m1), *SNAIL1* (Hs00195591_m1), *SNAIL2* (Hs00950344_m1), *VIM* (Hs00185584_m1), and *ZEB1* (Hs01566410_m1) by Taqman Gene Expression Assays (Applied Biosystems, Waltham, MA, USA) using the *GAPDH* gene as reference. cDNA was performed by High Capacity cDNA (Thermo Fisher Scientific, Eugene, OR, USA) and RT-qPCR runs in a 7900HT Fast Real-Time PCR System (Applied Biosystems, Waltham, MA, USA).

### 2.11. Statistical Analysis

Data were reported as mean values ± SD in at least three replicates. MiRNA and mRNA expression data were obtained from SDS 2.4 software (Applied Biosystems, Waltham, MA, USA) and the relative expression analysis was performed by the 2^−∆∆Ct^ method [31].

The Shapiro–Wilk test and qqplot were used to confirm data normality and Levene’s test was used to evaluate the homogeneity of variance. The data were analyzed by Student’s *t*-test or two-way ANOVA using GraphPad Prism version 7 (La Jolla, CA, USA). *p*-values < 0.05 were considered statistically significant. 

## 3. Results

### 3.1. Manipulation of MiR-182-5p Expression Levels Differentially Affects Cell Proliferation and the Cell Cycle Phases in PC-3 and DU145 Cells

**Figure 1:**
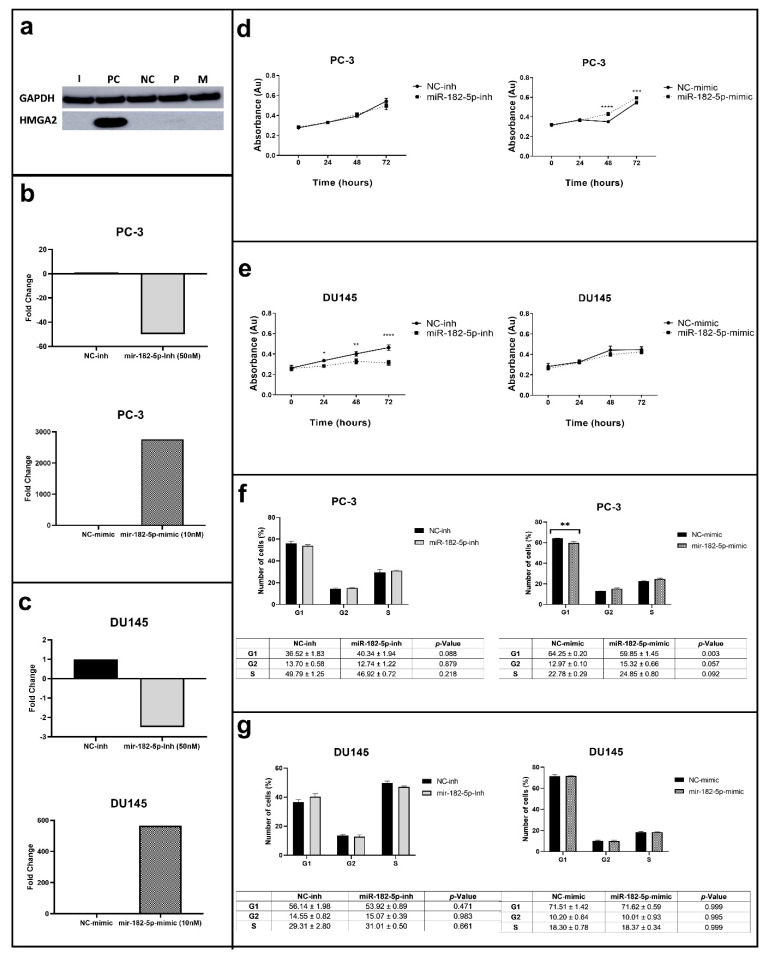
MiR-182-5p transfection, proliferation and cell cycle assays in the PC-3 and DU145 PCa cells. (**a**) HMGA2 (positive control for transfection) protein expression analysis; GAPDH used as loading control; (**b**,**c**) MiR-182-5p expression levels by RT-qPCR in the miR-182-5p transfected PC-3 and DU145 cells in relation to the NC, respectively; (**d**,**e**) Cell proliferation analysis in the PC-3 and DU145 transfected cells, respectively; (**f**,**g**) Cell cycle analysis in the PC-3 and DU145 transfected cells, respectively. I = miR-182-5p-inh, PC = Positive control; NC = Negative control; P = Parental; M = miR-182-5p-mimic. Statistically significance * *p* < 0.05; ** *p* < 0.01; *** *p* < 0.001; **** *p* < 0.0001.

The concentrations of the inhibitor (50 nM) and mimic (10 nM) assays used significantly reduced and increased the expression levels of miR-182-5p in the PCa cells studied, respectively (Figure 1a–c). The manipulation of the miR-182-5p expression levels differentially affected cell proliferation in the PC-3 and DU145 cells (Figure 1d). In the PC-3 cells, ectopic expression of the miR-182-5p increased cell proliferation (after 48 and 72 h), while its inhibition did not change cell proliferation rate when compared to the negative control (NC). On the other hand, in the DU145 cells, the inhibition of miR-182-5p led to the decrease of their proliferation rate after 24, 48, and 72 h; its ectopic expression did not cause any changes (Figure 1e).

In the PC-3 cells, the inhibition of miR-182-5p caused no effects in the cell cycle phases, however, its ectopic expression induced an increase in the number of cells in the G2/M phase and a reduction in the G1 phase when compared to the NC (Figure 1f). No statistically significant effect was observed in the distribution of the number of cells in the cell cycle phases of the miR-182-5p transfected DU145 cells (Figure 1g).

### 3.2. Manipulation of MiR-182-5p Expression Levels Differentially Affects Cell Adhesion and Migration in PC-3 and DU145 Cells 

**Figure 2:**
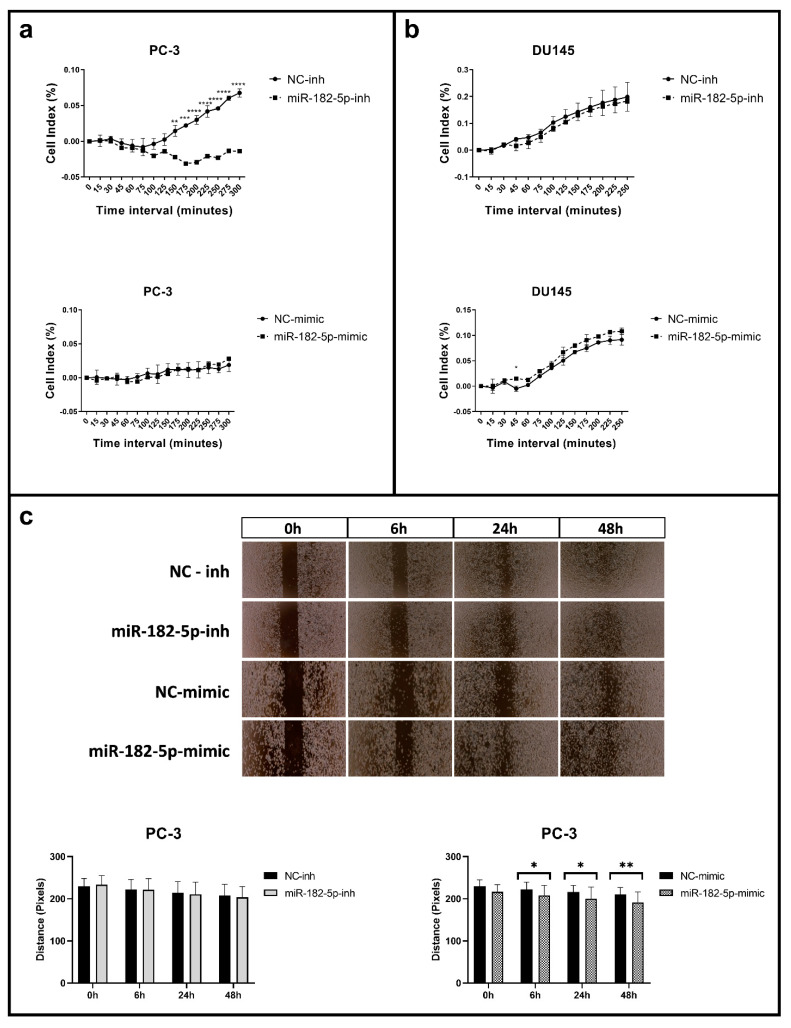
Cell adhesion and migration assays in the miR-182-5p transfected PCa cells. (**a**,**b**) Real time adhesion assay in the miR-182-5p transfected PC-3 and DU145 cells, respectively; (**c**) Wound healing cells images and gap distance measurements in the PC-3 miR-182-5p transfected cells at 0, 6, 24 and 48 h. NC = negative control; inh = inhibitor. Statistically significance * *p* < 0.05; ** *p* < 0.01; *** *p* < 0.001; **** *p* < 0.0001.

As in the proliferation and the cell cycle assays, cell adhesion and migration were differentially affected upon manipulation of the miR-182-5p expression levels in the PC-3 and DU145 cells. In the PC-3 cells, the inhibition of the miR-182-5p expression levels caused a delay in the time of the cells to adhere to the plate. This effect was not reversed, however, with the ectopic expression of miR-182-5p (Figure 2a). In the DU145 cell line, no significant effect in cell adhesion was observed upon manipulation of miR-182-5p expression levels (Figure 2b).

The ectopic expression of miR-182-5p in the PC-3 cells caused a significant reduction of the wound gap (as measured by the distance of the gap between the two sides of the insert), reflected by the increase in cell migration; however, no effect in cell migration was observed in these cells upon miR-182-5p inhibition (Figure 2c). In the DU145 cell line, no changes in cell migration were observed upon manipulation of the miR-182-5p expression levels (data not shown). 

### 3.3. Ectopic Expression and Inhibition of MiR-182-5p in DU145 Cells Increases Resistance to Docetaxel and Abiraterone

**Figure 3:**
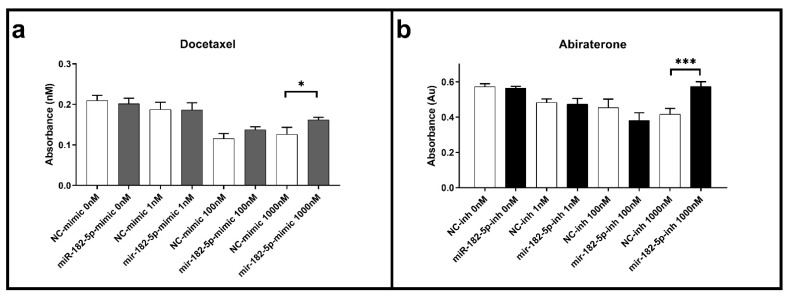
Docetaxel and abiraterone cell viability assays in the miR-182-5p transfected DU145 cells. (**a**) Cell viability in the docetaxel treated cells with ectopic expression of miR-182-5p; (**b**) Cell viability in the abiraterone treated cells transfected with miR-182-5p inhibitor. Drug exposure time of 72 h. NC = negative control; inh = inhibitor. Statistically significance * *p* < 0.05; *** *p* < 0.001.

Cell viability assays were performed using the MTT assay in the transfected PCa cells to docetaxel and abiraterone, common chemotherapeutic agents used to treat both primary and metastatic PCa patients. In the PC-3 transfected cells, no effects in the docetaxel and abiraterone cell viability were observed when compared to the NC for any of the concentrations and times of exposure tested (data not shown). In the DU145 cells, the ectopic expression of miR-182-5p led to a significant increase in cell viability when compared to the NC at 72 h in the maximum dose of docetaxel tested (1000 nM) (Figure 3a). No changes in cell viability were observed for this agent with the inhibition of miR-182-5p (data not shown). On the other hand, in the transfected cells with miR-182-5p inhibitor, a significant increase in cell viability was also observed with abiraterone treatment (Figure 3b); no significant effects to this agent were observed in the DU145 transfected cells with miR-182-5p mimic (data not shown)

### 3.4. MiR-182-5p Regulates mRNA Targets of EMT-Associated Functions

An initial KEGG enrichment pathway analysis of the miR-183 family revealed their common involvement in prostate cancer, and critical cancer signaling pathways, including adherens junctions, proteoglycans in cancer, Hippo, FoxO, PIK3/AKT, AMPK, and estrogen signaling pathways (Table 1). MiR-182-3p, miR-183-3p, and miR-96-3p were not accounted for in this analysis considering that none of their targets were experimentally validated.

**Figure 4:**
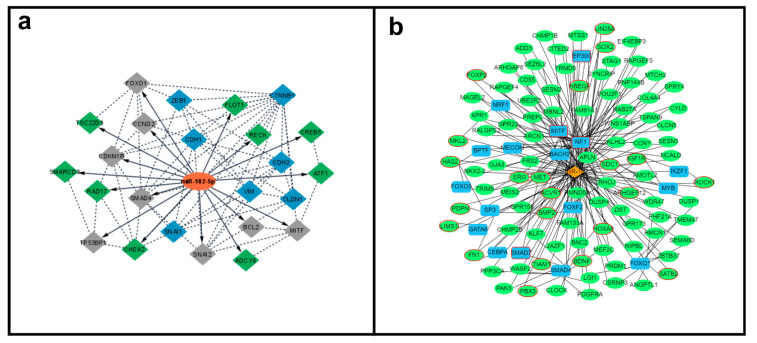
MiR-182-5p/mRNA target interaction networks. (**a**). MiR-182-5p (orange color) interaction with mRNA experimentally validated targets. Gray color = direct EMT associated miR-182-5p mRNA targets, green color = general miR-182-5p mRNA targets and blue color: EMT expression genes evaluated. Solid line = miR-182-5p mRNA targets, dashed lines miR-182-5p and mRNA targets interactions (Cytoscape v.3.8.0). (**b**). Regulatory relationship of miR-182-5p (orange color) with transcription factors (blue color), miR-182-5p mRNA targets (green color), and EMT genees (red circles) (EMTRegulome analysis, motif type 1, *p* < 0.05).

Following a comprehensive computational analysis, the miR-182-5p mRNA targets and their interaction were determined. The miRTarBase and TarBase databases identified 45 and 28 target genes, respectively. Only targets from strong experimentally validated assays were considered. The comparison of genes from these two databases resulted in 22 common miR-182-5p target genes (Supplementary Table S1). These selected targets were then “cross-checked” in the dbEMT and EMTRegulome databases, to identify the ones with potential association with the EMT process. The comparison of the 22 target genes with the 1184 genes with EMT function present in the dbEMT2 database, revealed nine gene targets involved in the EMT process (*BCL2*, *CCND2*, *CDKN1B*, FOXO1, *MITF*, *SATB2*, *SMAD4*, *SNAI2*, and *TP53BP1*). These target genes, together with other classical EMT markers (*CDH1*, *CDH2*, *CLDN1*, *CTNNB1*, *SNAI1*, *VIM*, and *ZEB1*) were evaluated for PPI analysis (Supplementary Figure S1), to further generate a miR-182-5p/EMT-mRNA target network (Figure 4a). Seven target genes showed no PPI, including one gene with EMT function (*SATB2*).

Independently, the EMTRegulome analysis (using motif type 1 and *p* < 0.05), revealed a complex regulatory relationship of miR-182-5p with 123 target genes, 22 genes related to the EMT process, 13 transcription factors (TF), and four TF with EMT function reinforcing the role of miR-182-5p in regulating the EMT process (Figure 4b).

### 3.5. Overexpression of MiR-182-5p in PC-3 Cells Leads to the Increase of EMT Promoter Markers’ Expression

**Figure 5:**
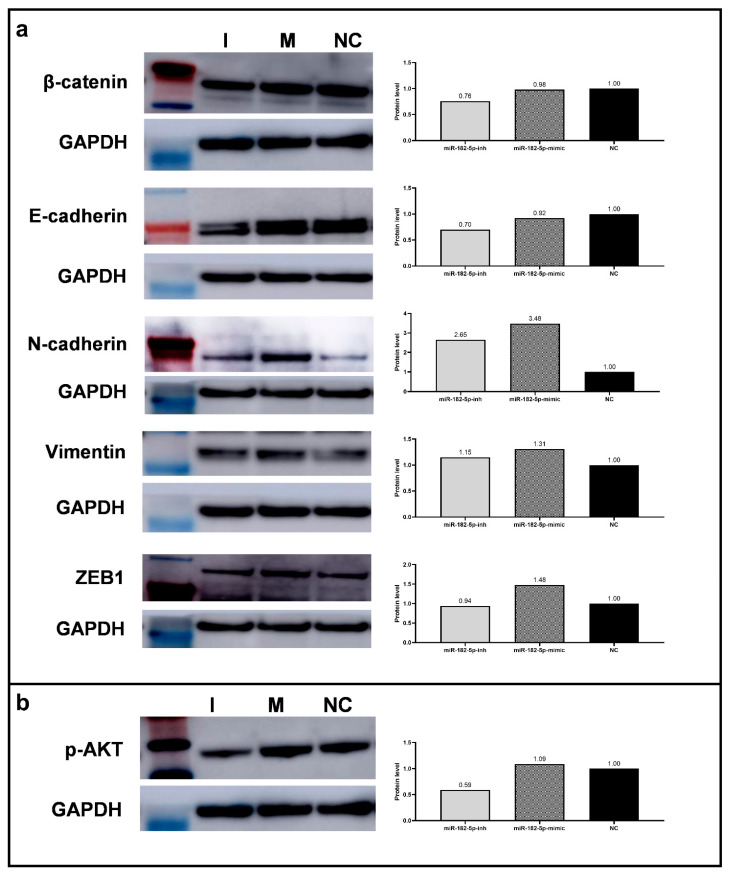
Protein and mRNA expression levels of EMT associated markers in the miR-182-5p transfected PC-3 cells. (**a**) Expression analysis of EMT markers (β-Catenin, E-Cadherin, N-Cadherin, Vimentin, and ZEB1) by Western blot and protein levels’ quantification (Image J). (**b**) p-AKT expression analysis by Western blot and expression levels’ quantification (Image J). GAPDH was used as loading reference. NC = negative control; I = miR-182-5p-inh; M = miR-182-5p-mimic.

Considering the identification of miR-182-5p as a (direct and/or indirect) regulator of major EMT markers, as evidenced by the computational enrichment analysis above, we performed Western blot and RT-qPCR analysis in the PC-3 cells to determine whether alterations of its expression levels would directly affect the expression of selected EMT markers and whether it could be one of the mechanisms by which miR-182-5p modulated the PCa phenotypes evaluated. The ectopic expression of miR-182-5p up-regulated the protein expression levels of the EMT promoter marker ZEB1, and the mesenchymal proteins N-Cadherin, and Vimentin, in relation to the NC. On the other hand, inhibition of miR-182-5p expression levels, down-regulated the expression of E-Cadherin and β-Catenin (Figure 5a). Similar results were obtained by RT-qPCR analysis for *CDH1*, *VIM*, and *ZEB1* gene expression in the transfected cells in relation to the NC (Table 2).

The additional markers evaluated for RT-qPCR, *SNAIL1*, and *SNAIL2* (a direct target of miR-182-5p), EMT repressors, were down-regulated upon miR-182-5p ectopic expression, while *CLDN1* (Claudin 1), a mesenchymal cell marker, was up-regulated (Table 2).

In addition, the expression levels of the protein p-AKT, considered a tumor progression marker, were also determined by Western Blot analysis after manipulation of the miR-182-5p expression levels in the PC-3 cells. Decreased expression levels of this protein were observed in the cells transfected with miR-182-5p inhibitor (Figure 5b).

## 4. Discussion

MiR-182-5p is determinant for the prognosis and tumor progression of PCa cells and impacts PCa cancer aggressiveness in in vitro and in vivo models [32]. However, the role of miR-182-5p in regulating PCa tumor phenotypes is not consistent. In this study, we evaluated the role of miR-182-5p in modulating tumor phenotypes in two androgen refractory cell lines, PC-3 and DU145. The lines’ selection was based on the major clinical challenge of tumor resistance to androgen-based therapy and on their large use as “classical” in vitro models of advanced and resistant prostate cancer. The functional assays using repressors and inducers of miR-182-5p expression showed mostly an oncomiR compatible mode of action of miR-182-5p in these PCa cells, however, its action varied according to the cell line and/or to the phenotype analyzed.

In the PC-3 cells, the ectopic expression of miR-182-5p significantly increased its proliferation rate. Consistently, flow cytometry analysis in these transfected cells showed a significant decrease in the number of cells in the G1 phase and a tendency for an increase in the number of cells in the S and G2 phases. On the contrary, in the DU145 cells, no effects were observed with the ectopic expression of miR-182-5p, but a significant decrease in cell proliferation was observed with miR-182-5p expression inhibition. In these cells, although not significant, it was observed an increase in the number of cells in the G1 phase, and a decrease in the number of cells in the S and G2 phases.

It is interesting to point out, however, that AR is known to upregulate the expression of p21CIP1, a well-known inhibitor of cyclin-dependent kinases, in prostate cancer cell lines, including the ones tested in this study [33]. Additional assays would be interesting to measure the expression levels of p21 in the transfected cells.

This oncomiR mode of action in cell proliferation was also observed in other PCa studies. Hirata et al. [14] and Bai et al. [18], in an analysis of the same PCa cells as this study, reported reduced cell proliferation with the inhibition of miR-182-5p expression. Reversely, Yao et al. [12], showed that miR-182-5p overexpression promoted the growth and progression of prostate cancer tumors in in vitro and in vivo models. Similar effects in cell proliferation of miR-182-5p transfected cells were observed in other cell tumor models, such as from melanomas [34], gliomas [35], hepatocellular [36], breast [17], and colorectal cancer cells [37]. In osteosarcomas [38], gastric cancer [39], and other colorectal cancer cells [40], however, an inverse effect was observed, indicating a tumor suppressor mode of action for miR-182-5p.

In the PC-3 cells, the cell proliferation effects regulated by miR-182-5p were consistent with the adhesion and migration phenotypes. Inhibition of miR-182-5p, led to the reduction of the capacity of the cells to adhere to the solid substrate, as demonstrated by the real-time adhesion assays; and its ectopic expression induced a higher migratory rate as compared to the negative control. These phenotypes were not observed in the same transfected cells systems, as could be explained by the differences in inhibiting and overexpressing the miR-182-5p endogenous levels and/or by the impact of the miR-182-5p manipulation in downstream regulation of cofactors, either co-activators or co-repressors of the AR signaling [41], that can affect the distinct tumor phenotypes. However, it confirms the association of cell adhesion with cell proliferation and migration capabilities in tumor cells [42,43] and the direct regulation of these phenotypes by miR-182-5p. Similar findings in cell adhesion, migration, and invasion were observed in other PCa cells studies [12,14,16,18,44,45] and in other tumor models [32,46,47]. Altogether, these results support the oncomiR function of miR-182-5p in regulating cell proliferation, adhesion, and migration of PCa cells, which largely determines their tumorigenic capacity and the observed unfavorable outcome in PCa clinical cases that present with miR-182-5p overexpression [8,9,10,12,13,14,15,45].

The cell viability analysis of miR-182-5p in our study showed distinct modes of action according to the chemotherapeutic agent tested. In DU145 cells treated with docetaxel, the ectopic expression of miR-182-5p led to a significant increase in cell viability when compared to the negative control. Docetaxel is the first line of treatment for patients with advanced prostate cancer patients, including the ones with the metastatic castration-resistant form of the disease [48,49,50]. However, approximately 30% of the patients present primary resistance to this drug, and over time even the ones that initially responded develop resistance [51,52]. MiR-182-5p has been associated with chemoresistance in several tumor types [53,54,55,56], however, to the best of our knowledge there are no reports on its direct association to docetaxel resistance in PCa cells. Nonetheless, miR-183, another member of the miR-183-96-182 cluster, was reported to be overexpressed in PCa [48] and associated with docetaxel resistance [51,57]. MiR-96-5p was also observed to be associated with docetaxel resistance in several PCa cell lines, including the PC-3 [58]. Considering the observed coordinated expression of the miRNAs composing the highly conserved miR-183 family and their cooperation in regulating cancer-associated signaling pathways [59], it is expected that miR-182-5p, as the other cluster’ members, also plays a pivotal role in docetaxel resistance. 

Cell viability to abiraterone acetate, an androgen blocker that acts by inhibiting the CYP17A1 enzyme [60] and a second-line treatment for metastatic PCa patients [61], was also tested in the PCa cells of this study. As per the docetaxel treatment, in the PC-3 no changes in cell viability were observed. In the DU145 cells, significant changes in the viability of abiraterone were observed at the maximum dose at 72 h. However, unexpectedly with the inhibition of miR-182-5p expression levels, it was observed an increase in cell resistance to this agent, which is consistent with a suppressor-like mode of action. Interestingly, among the several mRNAs predicted targets of miR-182-5p is the *ACTIVIN A* gene (also known as *INHBA* gene), a member of the TGF-beta family and an antagonist of CYP17A1 expression [62]. Therefore it is possible that the lower expression of miR-182-5p, caused by its inhibition, will not repress ACTIVIN A expression, which in turn will repress CYP17A1 [63] not allowing the full action of abiraterone and leading to cell resistance, as observed here. In this case, cells treated with CYP17A1 blockers, such as abiraterone, will present a less effect on cell viability when compared to cells that present high levels of this target protein. In addition, the repression of ACTIVIN 1, which positively regulates the aldosterone hormone production, increases the levels of this hormone which was shown to be associated with castration-resistant tumors [51,64].

The functional enrichment analysis of miR-182-5p, and the other cluster members, revealed KEGG pathways in prostate cancer and others commonly involved in cell signaling cascades that mediate cell proliferation, cell survival, and cell cycle control, such as PI3K/AKT and FoxO, which are cooperating pathways that play essential roles in PCa disease progression [65]. In addition, pathways directly related to the EMT process and tumor progression were significantly identified, including adherens junctions, cytoskeleton regulation, ECM receptor interaction, and others. Several studies have shown that the EMT process is controlled post-transcriptionally by miRNAs [66,67] and a number of miRNAs, including miR-182-5p, have been identified to target multiple components of this process [67,68,69,70]. In fact, by conducting a search in the dbEMT and EMTRegulome databases, which contain over 1000 experimentally verified EMT-related genes from the literature [27,28], nine (*BCL2, CCND2, CDKN1B, FOXO1, MITF, SATB2, SMAD4, SNAIL2*, and *TP53BP1*) out of the 22 experimentally validated miR-182-5p mRNA targets identified were involved in the EMT process. These mRNA targets were identified to form a complex regulatory relationship with miR-182-5p, composed of 123 target genes, 22 genes related to the EMT process, 13 transcription factors (TF), and four TF with EMT function. To directly determine whether alterations in the EMT markers could be one of the mechanisms by which miR-182-5p modulated the tumor phenotypes in the PCa cells, we evaluated the protein and mRNA expression levels of seven EMT markers (*CTNNB1, CDH1, CLDN1, SNAIL1, SNAIL2, VIM*, and *ZEB1*) in PC-3 transfected cells. The ectopic expression of miR-182-5p led to the up-regulation of the protein expression levels of ZEB1, an EMT promoter marker, and in the N-Cadherin and Vimentin, EMT-associated mesenchymal markers [42,71,72]. On the other hand, in cells transfected with miR-182-5p inhibitor, a down-regulation of the expression of the nuclear β-Catenin, another EMT-mesenchymal associated protein [66], was observed. Interestingly, however, this inhibition also led to decreased levels of E-Cadherin, an EMT-epithelial associated protein [66]. This can indicate, as observed in the increased cell viability to abiraterone upon miR-182-5p inhibition, a distinct mode of action (tumor suppressor-like) of miR-182-5p in regulating E-Cadherin or an indirect mechanism through the regulation of other EMT downstream targets. The latter can be exemplified by the “sequestration” of β-Catenin in cells with high expression of E-Cadherin to avoid its action as a DNA binding protein that activates the Wnt signaling pathway cascade of genes, well known to be associated with cancer development [66]. MiR-182-5p ectopic expression also led to the decreased expression levels of the EMT repressors, *SNAIL1*, and *SNAIL2* genes, the latter a direct miR-182-5p target gene. This finding corroborates with Qu et al. [73] that reported an association of miR-182-5p with cell growth by repressing *SNAIL2* expression in PCa cells. Other studies have shown that the downregulation of *SNAIL2* expression is frequent in PCa tissue [74,75]. On the other hand, the mRNA levels of *CLDN1*, which codify Claudin1, which were observed up-regulated in our study upon inhibition of miR-182-5p, have been reported to be upregulated or downregulated in tumor cells [76].

Finally, we also assessed whether the tumorigenicity of the PCa cells mediated by miR-182-5p could be due to alterations in the p-AKT protein levels, a marker that is reported to be directly associated with PCa cell invasion and tumor progression [77,78]. Indeed, we observed that cells with miR-182-5p inhibition presented down-regulation of the p-AKT levels. Similar effects of miR-182-5p were observed in multiple myelomas [79], showing its close relation with the p-AKT marker. The PIK3/AKT pathway, one of the pathways significantly observed in KEGG analysis of miR-182-5p, has a critical role in regulating the EMT process in several types of cancer, including PCa [80,81], by regulating several transcription factors, as well as, by miRNA regulation [77,82,83].

In conclusion, our data support the role of miR-182-5p in regulating tumor aggressive phenotypes in PCa androgen-refractory cells, in a compatible oncomiR mode of action, by targeting EMT-associated pathways.

## Figures and Tables

**Table 1:** Fifteen selected significant KEGG pathways of the miR-183 family members (miR-182 higlighted in bold) (presented by *p*-value).

KEGG Pathway	*p*-Value	#Genes	MiR-183 Cluster’ Members
Adherens junction	3.67 × 10^−14^	34	**miR-182**, miR-183, miR-96
Fatty acid biosynthesis	2.69 × 10^−12^	3	**miR-182**
Hippo signaling pathway	3.69 × 10^−8^	37	**miR-182**, miR-183
Proteoglycans in cancer	2.36 × 10^−7^	61	**miR-182**, miR-183, miR-96
Prostate cancer	3.03 × 10^−7^	38	**miR-182**, miR-183, miR-96
Cell cycle	3.86 × 10^−6^	40	**miR-182**, miR-96
FoxO signaling pathway	4.96 × 10^−6^	48	**miR-182**, miR-183, miR-96
Estrogen signaling pathway	7.15 × 10^−5^	30	**miR-182**, miR-183, miR-96
p53 signaling pathway	0.000103754	27	**miR-182**, miR-96
Regulation of actin cytoskeleton	0.000843268	27	miR-183
AMPK signaling pathway	0.001045423	39	**miR-182**, miR-96
Pathways in cancer	0.002560086	89	**miR-182**, miR-183
PI3K-Akt signaling pathway	0.003008112	33	miR-96
ECM-receptor interaction	0.0278908	8	miR-96
Axon guidance	0.03842599	14	miR-183

**Table 2:** Expression level of EMT gene markers in the miR-182-5p transfected PC-3 cells by RT-qPCR.

Protein	Gene	Fold-Change		
		MiR-182-5p-inh	MiR-182-5p-mimic	NC
β-catenin	*CTNNB1*	* 1.7	**1.5**	1.00
E-cadherin	*CDH1*	0.8	1.1	1.00
Vimentin	*VIM*	1.0	1.1	1.00
ZEB1	*ZEB1*	0.7	1.0	1.00
SNAIL1	*SNAIL1*	1.0	0.8	1.00
SLUG	*SNAIL2*	1.2	**0.4**	1.00
Claudin 1	*CLDN1*	0.9	1.3	1.00

* In bold, values of FC considered significant: >1.5 for up-regulation and <0.5 for down-regulation of the miR-182-5p.

## Data Availability

Not applicable.

## Supplementary Materials

The following supporting information can be downloaded at: https://www.mdpi.com/article/10.3390/biom12020187/s1, Table S1: title Genes validated by strong methods; Figure S1: Protein-protein interaction (PPI) analysis. Interaction of miR-182-5p target genes (validated by strong experimental methods) identified by the intersection of miRTarBase and TarBase v8 databases. The genes associated with EMT function (*CDH1, CDH2, CLDN1, CTNNB1, ZEB1, SNAI1, SNAI2, VIM*), evaluated in this study, were also added to the network.
