# Supplementary material for: MiR-182-5p Modulates Prostate Cancer Aggressive Phenotypes by Targeting EMT Associated Pathways
Source: Biomolecules. 2022 Jan 22;12(2):187. doi: 10.3390/biom12020187 (PMC8961520; doi:10.3390/biom12020187)
Supplement: Supplementary file 1 [file biomolecules-12-00187-s001.zip › biomolecules-1520410-supplementary/biomolecules-1520410-supplementary.pdf]

Supplementary Table S1: Genes validated by strong methods*

miR-182-5p		
miRTarBase (A)	TarBase v.8 (B)	Intersection AxB
ADCY6	*ADCY6*	*ADCY6*
ANUBL1	*ATF1*	*ATF1*
ARRDC3	*BCL2*	*BCL2*
ATF1	*BRCA1*	*CCND2*
BARD1	*CCND1*	*CDKN1B*
BCL2	*CCND2*	*CHEK2*
BDNF	*CDK4*	*CLOCK*
CADM1	*CDKN1B*	*CREB5*
CCND2	*CHEK2*	*FLOT1*
CDKN1A	*CLOCK*	*FOXF2*
CDKN1B	*CREB5*	*FOXO1*
CHEK2	*E2F1*	*MITF*
CHL1	*FLOT1*	*MTSS1*
CLOCK	*FOXF2*	*PFN1*
CREB1	*FOXO1*	*RAD17*
CREB5	*MITF*	*RECK*
CYLD	*MTSS1*	*SATB2*
FBXW7	*NFKBIB*	*SMAD4*
FGF9	*PFN1*	*SMARCD3*
FLOT1	*RAD17*	*SNAI2*
FOXF2	*RECK*	*TP53BP1*
FOXO1	*SATB2*	*TSC22D3*
FOXO3	*SMAD4*	
GSK3B	*SMARCD3*	
LRRC4	*SNAI2*	
MITF	*TP53BP1*	
MTSS1	*TSC22D3*	
NDRG1	*ZFAND4*	
NTM		
PDCD4		
PFN1		
PTEN		
RAD17		
RARG		
RECK		
SATB2		
SMAD4		
SMARCD3		
SNAI2		
TCEAL7		
THBS1		
TP53BP1		
TP53INP1		
TSC22D3		
ULBP2		

*Luciferase; Western Blot; RT-qPCR

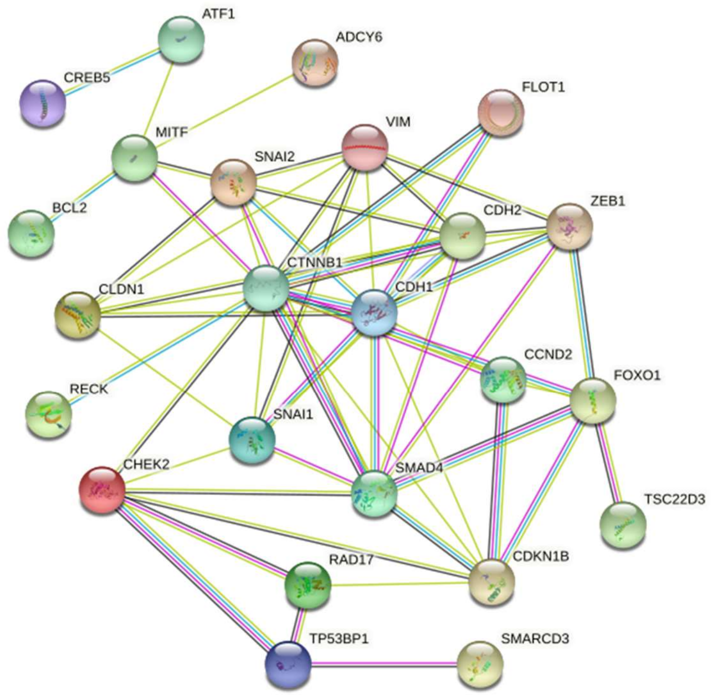

Supplementary Figure S1. Protein-protein interaction (PPI) analysis. Interaction of miR-182-5p target genes (validated by strong experimental methods) identified by the intersection of miRTarBase and TarBase v8 databases. The genes associated with EMT function (*CDH1*, *CDH2*, *CLDN1*, *CTNNB1*, *ZEB1*, *SNAI1*, *SNAI2*, *VIM*), evaluated in this study, were also added to the network.
